# Supplementary material for: Mutations in PCYT2 disrupt etherlipid biosynthesis and cause a complex hereditary spastic paraplegia
Source: Brain. 2019 Oct 22;142(11):3382–97. doi: 10.1093/brain/awz291 (PMC6821184; doi:10.1093/brain/awz291)
Supplement: awz291_Supplementary_Data [file awz291_supplementary_data.zip › awz291-Suppl_data/Supplementary_Data4.zip!/Supplementary information 4 - plasma lipidomics.zip!/Plasma lipidomics/Control vs Patient3b/Statistical plots and data/Heatmap sorted on VIP-score.pdf]

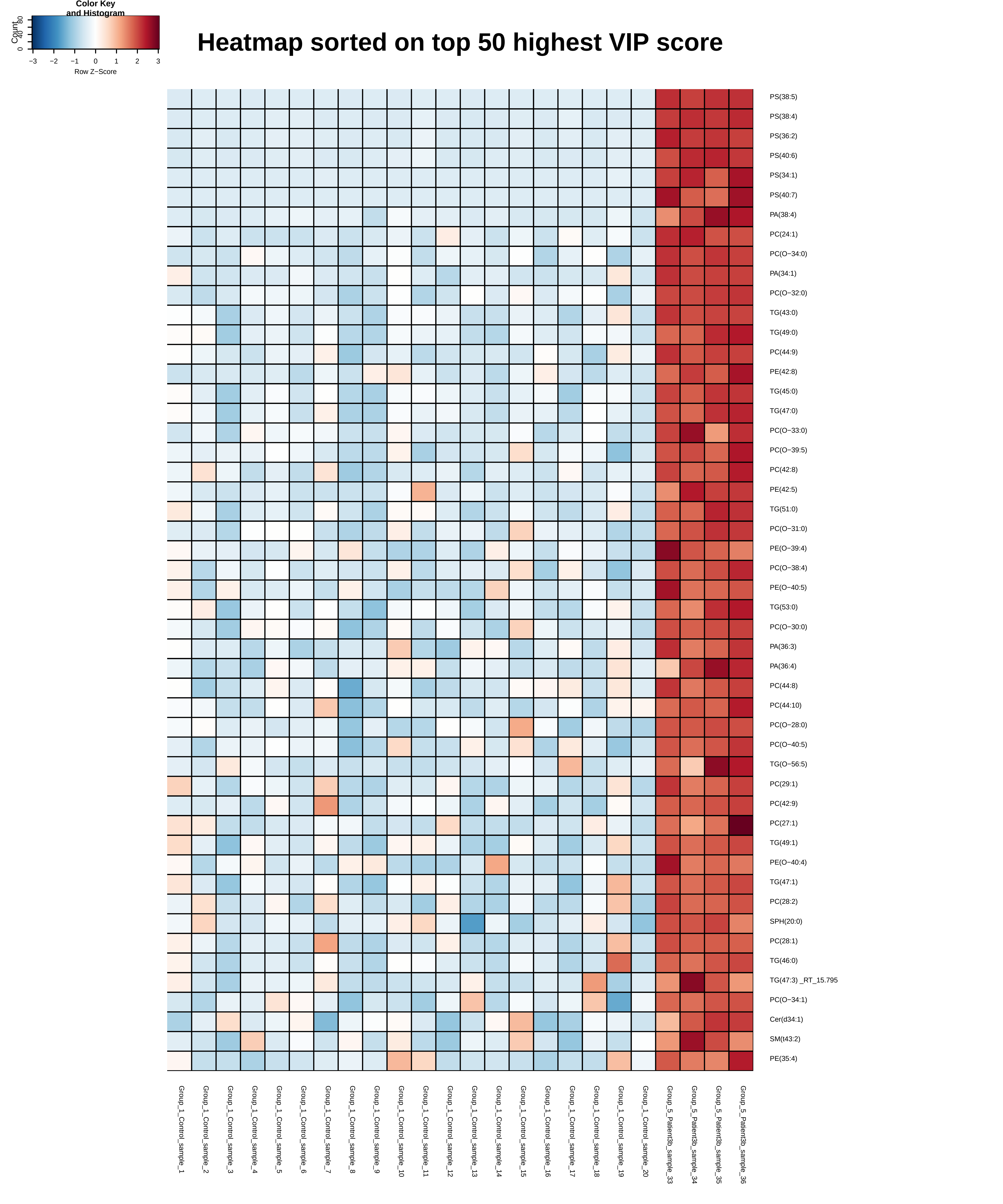

Color Key
and Histogram
Count
0
40
80
−3
−2
−1
0
1
2
3
Row Z−Score
Heatmap sorted on top 50 highest VIP score
PS(38:5)
PS(38:4)
PS(36:2)
PS(40:6)
PS(34:1)
PS(40:7)
PA(38:4)
PC(24:1)
PC(O−34:0)
PA(34:1)
PC(O−32:0)
TG(43:0)
TG(49:0)
PC(44:9)
PE(42:8)
TG(45:0)
TG(47:0)
PC(O−33:0)
PC(O−39:5)
PC(42:8)
PE(42:5)
TG(51:0)
PC(O−31:0)
PE(O−39:4)
PC(O−38:4)
PE(O−40:5)
TG(53:0)
PC(O−30:0)
PA(36:3)
PA(36:4)
PC(44:8)
PC(44:10)
PC(O−28:0)
PC(O−40:5)
TG(O−56:5)
PC(29:1)
PC(42:9)
PC(27:1)
TG(49:1)
PE(O−40:4)
TG(47:1)
PC(28:2)
SPH(20:0)
PC(28:1)
TG(46:0)
TG(47:3) _RT_15.795
PC(O−34:1)
Cer(d34:1)
SM(t43:2)
PE(35:4)
Group_1_Control_sample_1
Group_1_Control_sample_2
Group_1_Control_sample_3
Group_1_Control_sample_4
Group_1_Control_sample_5
Group_1_Control_sample_6
Group_1_Control_sample_7
Group_1_Control_sample_8
Group_1_Control_sample_9
Group_1_Control_sample_10
Group_1_Control_sample_11
Group_1_Control_sample_12
Group_1_Control_sample_13
Group_1_Control_sample_14
Group_1_Control_sample_15
Group_1_Control_sample_16
Group_1_Control_sample_17
Group_1_Control_sample_18
Group_1_Control_sample_19
Group_1_Control_sample_20
Group_5_Patient3b_sample_33
Group_5_Patient3b_sample_34
Group_5_Patient3b_sample_35
Group_5_Patient3b_sample_36
